# A/H1N1 infection: immunological parameters in ICU patients

**DOI:** 10.1186/cc10620

**Published:** 2012-03-20

**Authors:** I Zykova, P Sedlák, T Zajíc, A Vitouš, F Stejskal

**Affiliations:** 1 Regional Hospital Liberec, Czech Republic

## Introduction

The outbreak of influenza A/H1N1 2009 had influenced ICUs all over the world. In the season 2009/10 we admitted to intensive care 13 patients with A/H1N1 infection in our regional hospital. In the next season 2010/11 another outbreak of A/H1N1 infection was predicted. We decided to study the immunological profiles of these patients and its development in time.

## Methods

We conducted a prospective study on patients admitted to our hospital with A/H1N1 infection in the season 2010/11. The diagnosis was confirmed by RT-PCT from nasopharyngeal smear or bronchoalveolar lavage in all patients. Immunological parameters (leukocyte count, lymphocyte count, CD19, CD4, CD8, immunoregulatory index, NK cells) were analysed on admission and 3 weeks after admission.

## Results

In season 2010/11 only six patients with a confirmed A/H1N1 infection required admission to intensive care (47% of all patients with a confirmed A/H1N1 infection admitted to our hospital). All patients required ventilation. Median APACHE II score was 18.2. Median ICU stay was 18.5 days. Median number of ventilator days was 14. No patient died, both 28-day and 3-month mortality was 0%. Total leukocyte count was without substantial differences, but there was a prominent lymphopenia at the time of admission (0.05 to 0.22% of total leukocyte count) as has been described in similar studies. All lymphocyte populations were decreased but a most prominent decrease was in CD4 (T-helpers) and CD8 (T-suppressors), CD19 (B-lymphocytes) and NK cells were less decreased. Comparison of the admission sample and the second sample taken 21 days after admission: both CD4 and CD8 were most decreased at admission, immunoregulatory index had a shift to positive values in the admission sample.

## Conclusion

Our small sample of intensive care patients with a confirmed A/H1N1 infection supports the scarce published data about the early immunological profile of these patients. All our patients had a prominent lymphopenia with a most significant decrease in CD4 and CD8 cells. Due to the number of patients in the season 2010/11 and the survival of all patients we could not analyse the relation of survival and the change in time of immunological profile in this unique and probably already extinct group of patients.

## References

[B1] Shapovalov KG Immunological and bacteriological monitoring of patients with pneumonia and influenza A/H1N1 infection Zh Mikrobiol Epidemiol Immunobiol 2011 1 79 82 21446172

[B2] Kim JE CD4^+^/CD8^+ ^T lymphocytes imbalance in children with severe 2009 pandemic influenzaA/H1N1 pneumonia Korean J Pediatr 2011 54 207 211 10.3345/kjp.2011.54.5.207 21829412 PMC3145905

